# Individualized discrimination of tumor recurrence from radiation necrosis in glioma patients using an integrated radiomics-based model

**DOI:** 10.1007/s00259-019-04604-0

**Published:** 2019-11-26

**Authors:** Kai Wang, Zhen Qiao, Xiaobin Zhao, Xiaotong Li, Xin Wang, Tingfan Wu, Zhongwei Chen, Di Fan, Qian Chen, Lin Ai

**Affiliations:** 1 Department of Nuclear Medicine, Beijing Tiantan Hospital, Capital Medical University, 119, West Road of South 4th Ring, Fengtai District, Beijing, China; 2 Department of PET/MR Advanced Application, GE Healthcare, Beijing, China

**Keywords:** Glioma, Radiomics, Recurrence, MRI, PET

## Abstract

**Purpose:**

To develop and validate an integrated model for discriminating tumor recurrence from radiation necrosis in glioma patients.

**Methods:**

Data from 160 pathologically confirmed glioma patients were analyzed. The diagnostic model was developed in a primary cohort (*n* = 112). Textural features were extracted from postoperative ^18^F-fluorodeoxyglucose (^18^F-FDG) positron emission tomography (PET), ^11^C-methionine (^11^C-MET) PET, and magnetic resonance images. The least absolute shrinkage and selection operator regression model was used for feature selection and radiomics signature building. Multivariable logistic regression analysis was used to develop a model for predicting tumor recurrence. The radiomics signature, quantitative PET parameters, and clinical risk factors were incorporated in the model. The clinical value of the model was then assessed in an independent validation cohort using the remaining 48 glioma patients.

**Results:**

The integrated model consisting of 15 selected features was significantly associated with postoperative tumor recurrence (*p* < 0.001 for both primary and validation cohorts). Predictors contained in the individualized diagnosis model included the radiomics signature, the mean of tumor-background ratio (TBR) of ^18^F-FDG, maximum of TBR of ^11^C-MET PET, and patient age. The integrated model demonstrated good discrimination, with an area under the curve (AUC) of 0.988, with a 95% confidence interval (CI) of 0.975–1.000. Application in the validation cohort showed good differentiation (AUC of 0.914 and 95% CI of 0.881–0.945). Decision curve analysis showed that the integrated diagnosis model was clinically useful.

**Conclusions:**

Our developed model could be used to assist the postoperative individualized diagnosis of tumor recurrence in patients with gliomas.

**Electronic supplementary material:**

The online version of this article (10.1007/s00259-019-04604-0) contains supplementary material, which is available to authorized users.

## Introduction

Glioma is the most common and aggressive malignant brain tumor in adults [1]. The accurate identification of tumor recurrence in patients with gliomas is crucial for selecting treatment strategies to provide better therapeutic management. Early and accurate postoperative knowledge of tumor recurrence can provide valuable information for determining adjuvant therapies.

Previous studies revealed that ^18^F-fluorodeoxyglucose (^18^F-FDG) [2, 3], ^11^C-methionine (^11^C-MET) [4], ^18^F-fluoroethyl-l-tyrosine (^18^F-FET) [5, 6], and ^11^C-choline [7] PET, along with MRI, can differentiate between tumor recurrence and radiation necrosis with various levels of diagnostic efficiencies [8, 9]. However, conventional hybrid PET/MRI studies did not fully perform deep mining of the intrinsic features of the images, which could be further investigated using advanced methodology in a larger cohort [8–11].

Radiomics has attracted increased attention in recent years as it has the potential to improve the accuracy of recurrence predictions in oncology [12–15]. The application of radiomics enables parallel investigation of multiple imaging features and enables high-throughput mining of quantitative image features from standard-of-care medical imaging to improve diagnostic, classification, prognostic, and predictive accuracy, providing a powerful tool in modern medicine [12, 16–18]. Therefore, the aim of this study was to develop and validate an integrated model that incorporated features from PET (with both ^18^F-FDG and ^11^C-MET) and MRI images, along with clinical risk factors for individual discriminating tumor recurrence from radiation necrosis in glioma patients.

## Materials and methods

### Patients

For this retrospective analysis, ethical approval was obtained, and the informed consent requirement was waived by our institutional reviewing board. Selection of the cohort followed an evaluation of the institutional database in Beijing Tiantan Hospital for medical records from April 2015 to March 2018 to identify patients with cerebral gliomas who underwent surgical resection. The inclusion and exclusion criteria are as follows: inclusion criteria: (1) patients who underwent surgery for cerebral gliomas, (2) pathologically confirmed cerebral gliomas, (3) postoperative MRI (including contrast-enhanced T1-weighted imaging) and PET (including both ^18^F-FDG and ^11^C-MET PET) were performed (the time between MRI and PET scans was less than 2 weeks), (4) postoperative radiotherapy received with or without chemotherapy, and (5) interview or telephone follow-up information available; exclusion criteria: (1) preoperative central nervous system disease of other kinds, (2) unknown histological grade, and (3) loss of contact post-operation/patient did not return for postoperative procedures. Those patients who satisfied each inclusion or exclusion criterion were identified for the whole cohort and were further assigned to either the primary cohort or validation cohort randomly.

### Treatment and follow-up

Gross total resection (GTR) was defined as there was no visible contrast enhancement on postoperative MR images within 48 h of surgery for contrast-enhanced tumors, or all the abnormal hyperintense changes on preoperative MR images for tumors not demonstrating contrast enhancement [19]. The postoperative adjuvant treatment was radiation therapy alone or concomitant temozolomide administration with fractionated radiotherapy, followed by up to six cycles of adjuvant temozolomide. Follow-up visit, MRI, and telephone interviews were conducted periodically after surgery with a minimum follow-up duration of 3 months after the completion of chemoradiotherapy. Tumor progression and radiation necrosis were defined according to the criteria in a previous study [20]. The overall follow-up duration of the study was 40 months, between May 2015 and September 2018. Accordingly, 118 patients (73 males and 45 females, mean age 44.48 ± 10.32 years with a range of 16 to 66 years) had tumor recurrence, and 42 patients (23 males and 19 females, mean age 44.74 ± 12.13 years with a range of 24 to 74 years) were identified as having radiation necrosis.

### Data assignment and MR and PET imaging

Of the 160 patients, 70% (112 patients) were assigned to the primary cohort by stratified sampling, including 83 cases of tumor recurrence and 29 cases of radiation necrosis; the remaining 30% (48 patients) were selected for the validation cohort with 35 cases of tumor recurrence and 13 cases of radiation necrosis.

MR images were obtained from GE 3.0T scanners (Genesis Signa and Signa HDe) and Siemens 3.0T scanners (Trio Tim and Verio). Post-contrast images were acquired immediately after injection of the contrast agent. The interval between contrast injection and the start of contrast-enhanced T1-weighted image acquisition was always 75–85 s. Postoperative MR scans for determining the extent of resection were performed within 72 h of this procedure, and the radiological parameters were maintained in accordance with the preoperative scans.

^18^F-FDG and ^11^C-MET PET images were acquired using a PET/CT scanner (Elite Discovery, GE Healthcare, USA) using a 5-mm axial resolution and full-width-at-half-maximum at the center of the field of view of 4 mm. Imaging data were reconstructed into 30 axial planes with a slice thickness of 5 mm and a 192 × 192 image matrix. All patients underwent ^18^F-FDG or ^11^C-MET PET scans according to the same protocol. ^18^F-FDG was intravenously injected at a dose of 3.7 MBq/kg and whole-brain image acquisition was started 60 min later. For ^11^C-MET PET, 555–740 MBq of ^11^C-MET was intravenously injected and whole-brain imaging was started 10 min later. Subjects were scanned in the supine position and instructed to remain completely quiet throughout the scanning procedure. The scanning times for both ^18^F-FDG and ^11^C-MET PET were 8–10 min. Postoperative PET scans were performed according to the onset of worsening symptoms of the patients after operation, and the time interval between ^18^F-FDG and ^11^C-MET PET was at least 2 days in order to eliminate the potential biological radiotracer crossover effect.

### Image pre-processing

PET and MR images with different resolutions were resampled and normalized to the same dimensions and grayscale level. The PET and MR images were not resampled simultaneously, but separately; and the resolution of PET images and MR images was not used. In order to minimize the loss of information, we separately perform image group feature extraction on them. The standardization process is carried out for the statistical analysis of the omics characteristics. For all 160 glioma patients, texture analysis was applied to their MR and PET (^18^F-FDG and ^11^C-MET) images using an in-house texture analysis software, called AnalysisKit (GE Healthcare, China). Contrast-enhanced T1WI, FLAIR, and PET (^18^F-FDG and ^11^C-MET) data were retrieved from the institution archive in Beijing Tiantan Hospital for the texture analysis herein. By using T1 contrast-enhanced (lesion showed contrast enhancement) or FLAIR (lesion without contrast enhancement) MR images as the reference modality of the delineation, the regions of interest (ROI) of the lesion for each slice of images were delineated manually by two experienced neuroradiologists. For each patient, the lesion mask (ROIs of the lesion) was combined to generate the final ROI for further texture analysis. The patient information was hidden during this process using ITK-SNAP software [21]. The image biomarker standardization initiative (IBSI) was regarded as reference and taken into consideration in most of the data processing, images feature, and biomarker selection procedure [22].

Two physicians performed ROI delineation for each patient and obtained two sets of radiomics features. In order to obtain a relatively stable integrated radiomics-based model, we calculated the relatively stable radiomics by calculating the intra-class coefficient correlation (ICC) index. A total of 1188 (396 × 3) imaging ensembles were obtained for the three sequences of FDG, MET, and MR images, and the characteristics of ICC > 0.8 were retained, which yielded a relative high inter-observer variability in the segmented tumor volume.

**Fig. 1:**
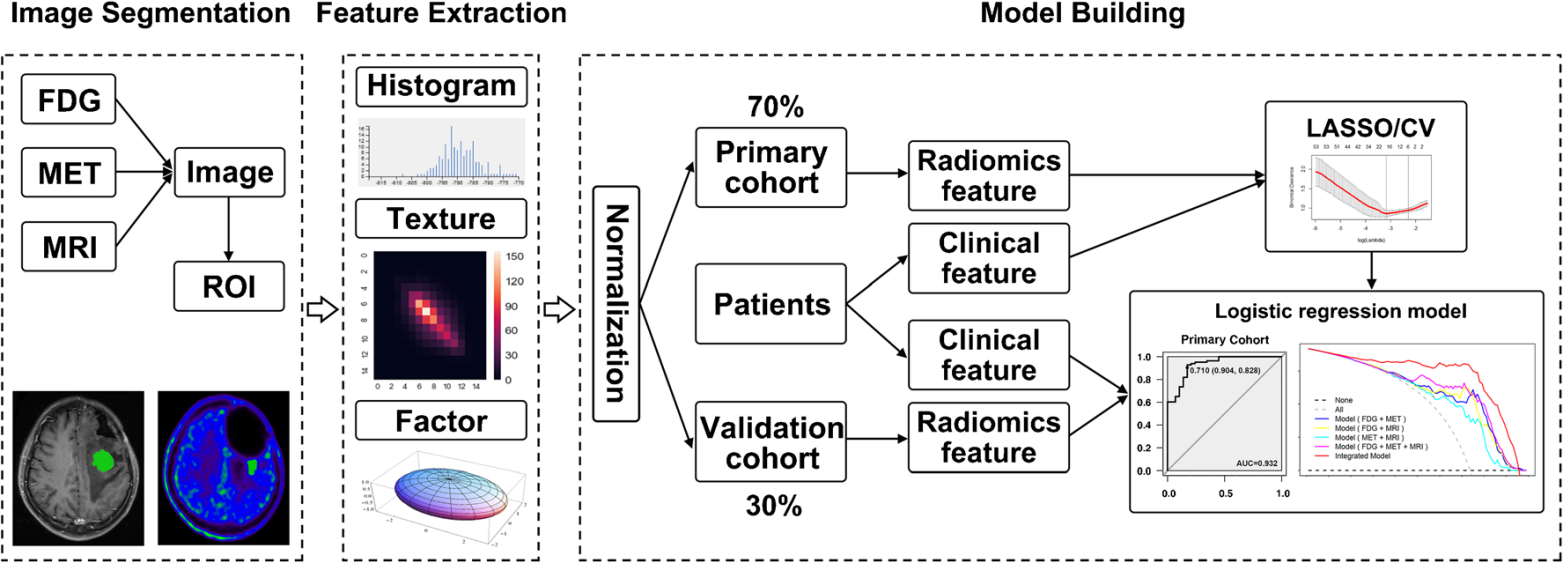
Schematic diagram showing the image analysis and model building processes. The abnormal signal region of ^18^F-FDG, ^11^C-MET, and MRI images was firstly segmented manually, followed by use of a feature extraction algorithm. Then, selection of image features and clinical factors was performed. Finally, the radiomics signature and patient features were applied for diagnostic evaluation to achieve personalized discrimination of tumor recurrence from radiation necrosis

The texture analysis–based 3D ROIs are reported in the Supplemental Data (Appendix 1). A flow chart of the analysis process used in the present study is shown in Fig. 1. All texture features were standardized and normalized with a regression model to remove error discrepancies introduced using different scanning instruments and methods.

### Feature selection and radiomics signatures

The least absolute shrinkage and selection operator (LASSO) method, which is suitable for the regression of high-dimensional data [23], was used to select the most useful predictive features from the primary data set. A radiomics score (rad-score) was calculated for each patient via a linear combination of selected features that were weighted by their respective coefficients. For the model with three imaging modalities (model_[FDG+MET+MRI]_), the performance of a specific radiomics signature for predicting tumor recurrence was first evaluated in the primary cohort and then confirmed in the validation cohort using an independent *t* test. Then, we compared the diagnostic efficiency of the radiomics signature between models with three modalities (model_[FDG+MET+MRI]_) and two modalities (model_[FDG+MET]_, model_[FDG+MRI]_, and model_[MET+MRI]_).

For all radiomics features, after obtaining 912 (FDG 303; MET 297; MRI 312) features with high consistency, since the features do not satisfy the normality, we use Spearman’s rank correlation coefficient redundancy analysis. The Spearman correlation coefficient takes a value of 0.9; that is, for all 912 features, a two-two correlation calculation is performed. When the coefficient *r* ≥ 0.9, the system will randomly delete one feature and retain another feature. In the end, there are 354 radiomics features; that is, the dimensions of the entire process feature range from 912 to 354.

### Integrated diagnosis model

The integrated model included patient features (age, gender, and body height and weight), contrast enhancement (+/−), the maximum of tumor-background ratio (TBR_max_) and the mean of tumor-background ratio (TBR_mean_) of both ^18^F-FDG and ^11^C-MET PET images, and tumor grade. Patient features and the radiomics signature were applied to develop an integrated diagnostic model for tumor recurrence using LASSO binary logistic regression analysis in the primary cohort. Similarly, an integrated score (int-score) was calculated for each patient via a linear combination of selected features that were weighted by their respective coefficients. Decision curve analysis was conducted to determine whether the model is clinically useful by quantifying the net benefits at different threshold probabilities in the validation cohort [24].

### Cross-validation

To improve the performance of the integrated model, a tenfold cross-validation of the model was carried out in the study. A lot of features were improved in the regularized L1 logistic regression with penalty term. As expressed in the following equation,

$$ L(w)=\kern0.5em \frac{1}{m}\sum \limits_{i=1}^m\left[\ln \left(1+\exp \left(\boldsymbol{\beta} \cdotp {x}^{(i)}\right)\right)-{y}^{(i)}\left(\boldsymbol{\beta} \cdotp {x}^{(i)}\right)\right]+\lambda \frac{1}{2}{\left\Vert \boldsymbol{\beta} \right\Vert}_1 $$

‖***β***‖_1_ was the penalty term, also expressed as ‖***β***‖_1_ =  |*β*_1_| + |*β*_2_| + … + |*β*_*p*_|. *L*(*w*) was the loss function.

For better performance of the integrated model, the best *λ* was obtained during the cross-validation procedure. Five independent sub-cohorts were divided in the training cohort, and four of which were applied for the model fitting; the other one sub-group was applied for the validation cohort. With 5 times repetition, each sub-group was treated as validation cohort. And finally, the *λ* was gained in the cross-validation set. And the results were displayed with such regularized L1 logistic regression [25]. The cross-validation procedure was carried out using R Studio software (version 1.2.1335).

### Statistical analysis

Statistical analysis was performed using R Studio software (version 1.2.1335) [26]. LASSO binary logistic regression was performed using the “glmnet” package. Multivariate binary logistic regression and diagnosis modeling were performed using the “stats” package. Decision curve analysis was performed using the “DecisionCurve” function.

The differences in patient features between patients with tumor recurrence and radiation necrosis in both the primary and validation cohorts were assessed by the independent sample *t* test or Mann-Whitney test according to the data distribution type. The chi-squared test was used to compare the significance of the differences between categorical variables. The same statistical analysis was performed to assess the difference between the two cohorts, where the tumor recurrence and radiation necrosis groups were evaluated separately. The diagnostic performance of models was evaluated using the receiver operating characteristic (ROC) curve. The statistical significance levels were all two-sided; the statistical significance was set at *p* < 0.05.

## Results

### Clinical characteristics

**Fig. 2:**
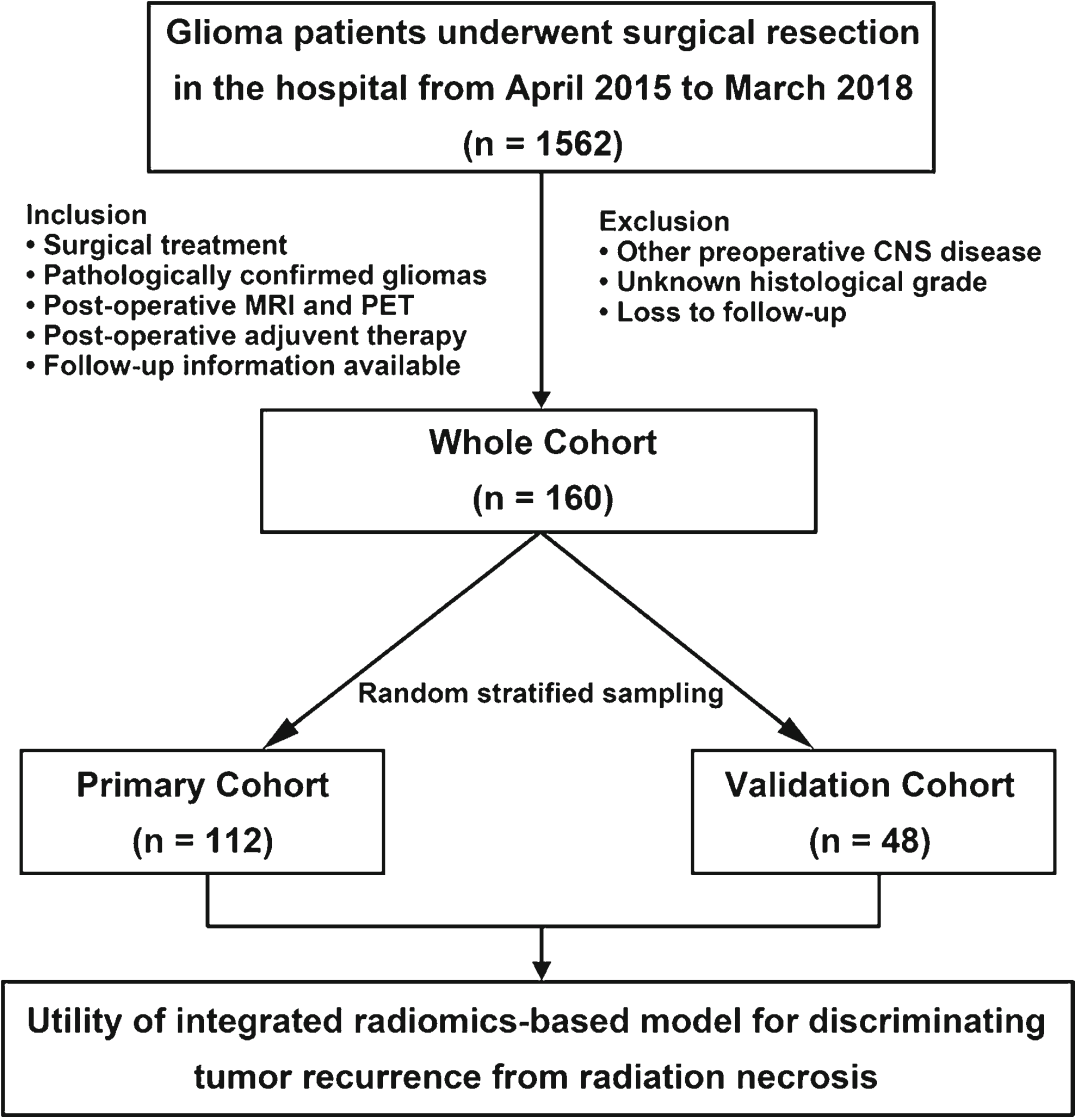
Flow chart of the selection of patients with cerebral gliomas who underwent surgical resection from April 2015 to March 2018. Based on the inclusion and exclusion criteria, a total of 160 glioma patients were enrolled in this study as the whole cohort and were further distributed randomly to either the primary cohort or validation cohort to explore and verify the discrimination performance of the model between tumor recurrence and radiation necrosis

From April 2015 to March 2018, there are 1562 patients with cerebral gliomas who underwent surgical resection in our institute. In total, 160 patients were identified for the whole cohort in the present study according to the inclusion and exclusion criteria, and were further assigned to either the primary cohort or validation cohort randomly (Fig. 2). The characteristics of the patients in the primary and validation cohorts are shown in Table 1. The rate of tumor recurrence in the primary and validation cohorts was 74.1% and 72.9%, respectively; this difference was not significant (*p* = 0.875). In addition, there were no significant differences in the patient features between the primary and validation cohorts, either within the tumor recurrence cohort or in the radiation necrosis cohort (Supplemental Tables 1-3). The difference between the rad-scores of the tumor recurrence and radiation necrosis patients in the primary cohort was significant (*p* < 0.001), which was also confirmed in the validation cohort (*p* < 0.001).

**Table 1:** Summary of the patient data in the primary and validation cohorts (*n* = 160) used in the study

	Primary cohort (*n* = 112)			Validation cohort (*n* = 48)		
	Tumor recurrence	Radiation necrosis	p*	Tumor recurrence	Radiation necrosis	p*
Age, mean ± SD (years)	43.87 ± 9.90	46.45 ± 11.61	0.251	45.94 ± 11.26	40.92 ± 12.84	0.193
Gender, no. (%)			0.878			0.571
Male	50 (60.2)	17 (58.6)		22 (62.9)	7 (53.8)	
Female	33 (39.8)	12 (41.4)		13 (37.1)	6 (46.2)	
MRI contrast enhancement			0.095			0.323
Yes	76 (91.6)	23 (79.3)		32 (91.4)	10 (76.9)	
No	7 (8.4)	6 (20.7)		3 (8.6)	3 (23.1)	
^18^F-FDG uptake						
TBR_max_	4.15 ± 2.41	2.28 ± 2.29	< 0.001	4.53 ± 2.96	2.32 ± 1.16	0.012
TBR_mean_	2.83 ± 1.38	1.54 ± 1.21	< 0.001	3.04 ± 1.75	1.63 ± 0.73	0.008
^11^C-methionine uptake						
TBR_max_	4.17 ± 2.62	1.74 ± 1.05	< 0.001	4.15 ± 1.53	2.05 ± 2.14	< 0.001
TBR_mean_	2.81 ± 2.12	1.23 ± 0.62	< 0.001	2.65 ± 1.07	1.33 ± 1.06	< 0.001
WHO grade, no. (%)			0.292			0.828
II	38 (45.8)	18 (62.1)		11 (31.4)	5 (38.4)	
III	21 (25.3)	6 (20.7)		14 (40.0)	4 (30.8)	
IV	24 (28.9)	5 (17.2)		10 (28.6)	4 (30.8)	
Radiomics score, mean ± SD	1.49 ± 0.52	0.19 ± 0.78	< 0.001	1.46 ± 0.55	0.43 ± 0.68	< 0.001
Integrated score, mean ± SD	2.27 ± 1.53	− 0.52 ± 0.95	< 0.001	2.20 ± 1.18	−0.09 ± 1.76	< 0.001

*p values were derived from the univariable association analysis between clinical variables

*SD*, standard deviation; *MRI*, magnetic resonance imaging; *FDG*, fluorodeoxyglucose; *TBR*, tumor-to-background ratio

**Fig. 3:**
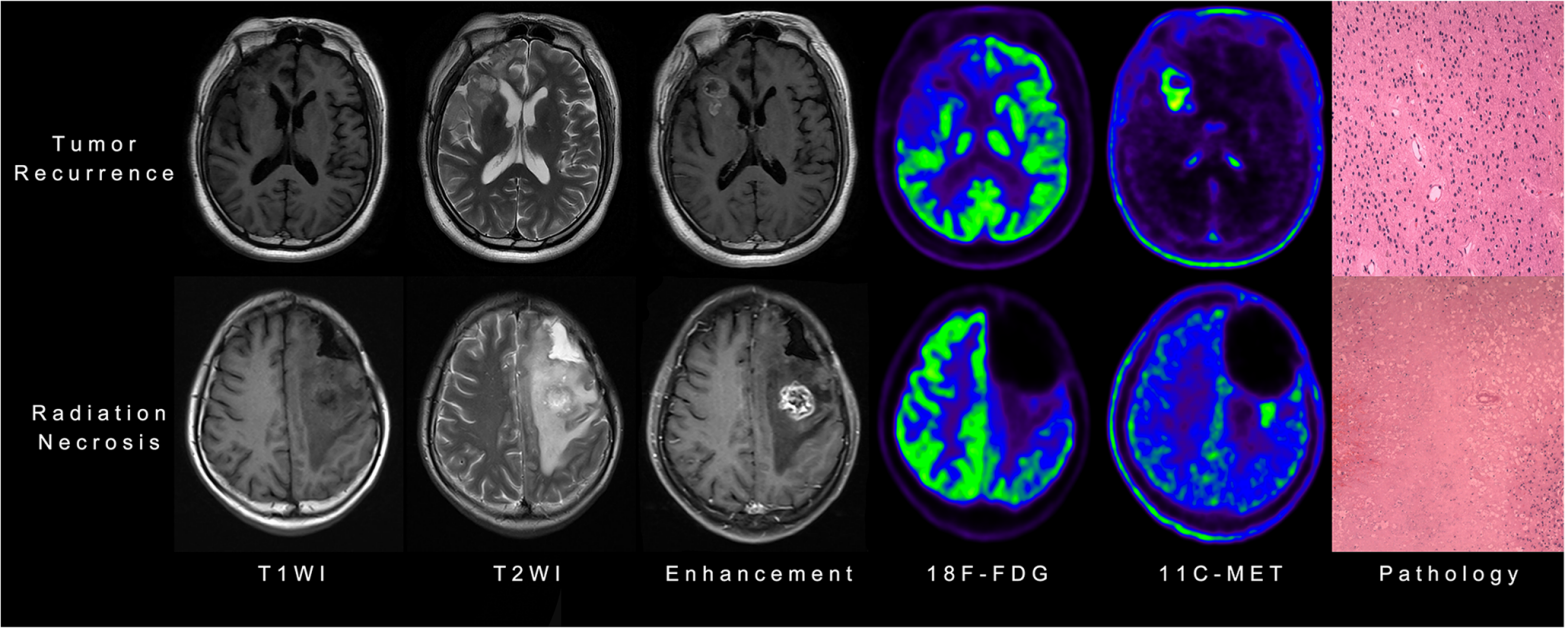
Representative MRI and PET images showing features of tumor recurrence and radiation necrosis, including images from T1-weighted (T1WI), T2-weighted (T2WI), and contrast-enhanced T1W1 MRI, ^18^F-fluorodeoxyglucose (^18^F-FDG) and ^11^C-methionine (^11^C-MET) PET, and pathological analyses

Representative MRI and PET images indicating the features of tumor recurrence and radiation necrosis are shown in Fig. 3. Of the texture features, 396 features were reduced to 20 potential features considering 112 patients in the primary cohorts (Supplemental Figure 1A). Calculation of the rad-score was performed using the formula shown as follows:

Radiomics score (rad-score) calculation

$$ {\displaystyle \begin{array}{c}\mathrm{Rad}\ \mathrm{score}=-1.161464\\ {}-0.111113\times \mathrm{Quantile}0.025\\ {}-0.187241\times \mathrm{RMS}\\ {}-0.154078\times \mathrm{ClusterProminence}\_\mathrm{AllDirection}\_\mathrm{offset}4\_\mathrm{SD}\\ {}+0.007201\times \mathrm{ClusterShade}\_\mathrm{angle}0\_\mathrm{offset}7\\ {}+0.266849\times \mathrm{ClusterShade}\_\mathrm{angle}135\_\mathrm{offset}7\\ {}+0.202809\times \mathrm{Correlation}\_\mathrm{AllDirection}\_\mathrm{offset}4\_\mathrm{SD}\\ {}+0.150674\times \mathrm{Correlation}\_\mathrm{angle}135\_\mathrm{offset}4\\ {}-0.119975\times C\mathrm{orrelation}\ \mathrm{angle}45\_\mathrm{offset}7\\ {}-0.014077\times \mathrm{HaralickCorrelation}\_\mathrm{AllDirection}\_\mathrm{offset}4\_\mathrm{SD}\\ {}+0.137885\times \mathrm{Inertia}\_\mathrm{AllDirection}\_\mathrm{offset}7\_\mathrm{SD}\\ {}-0.048716\times \mathrm{LongRunHighGreyLevelEmphasis}\_\mathrm{angle}135\_\mathrm{offset}7\\ {}+0.222189\times \mathrm{ShortRunLowGreyLevelEmphasis}\_\mathrm{angle}90\_\mathrm{offset}7\\ {}-0.025067\times \mathrm{RelativeDeviation}\\ {}-0.254328\times \mathrm{stdDeviation}\\ {}+0.102539\times \mathrm{GLCMEnergy}\_\mathrm{angle}45\_\mathrm{offset}7\\ {}+0.014467\times \mathrm{HaralickCorrelation}\_\mathrm{AllDirection}\_\mathrm{offset}1\_\mathrm{SD}\\ {}+0.111329\times \mathrm{HaralickCorrelation}\_\mathrm{AllDirection}\_\mathrm{offset}7\_\mathrm{SD}\\ {}-0.112513\times \mathrm{Sphericity}\\ {}-0.211177\times \mathrm{Correlation}\_\mathrm{angle}135\_\mathrm{offset}7\\ {}+0.074150\times \mathrm{HaralickCorrelation}\_\mathrm{AllDirection}\_\mathrm{offset}7\_\mathrm{SD}\end{array}} $$

### Diagnostic performance of radiomics signature

**Fig. 4:**
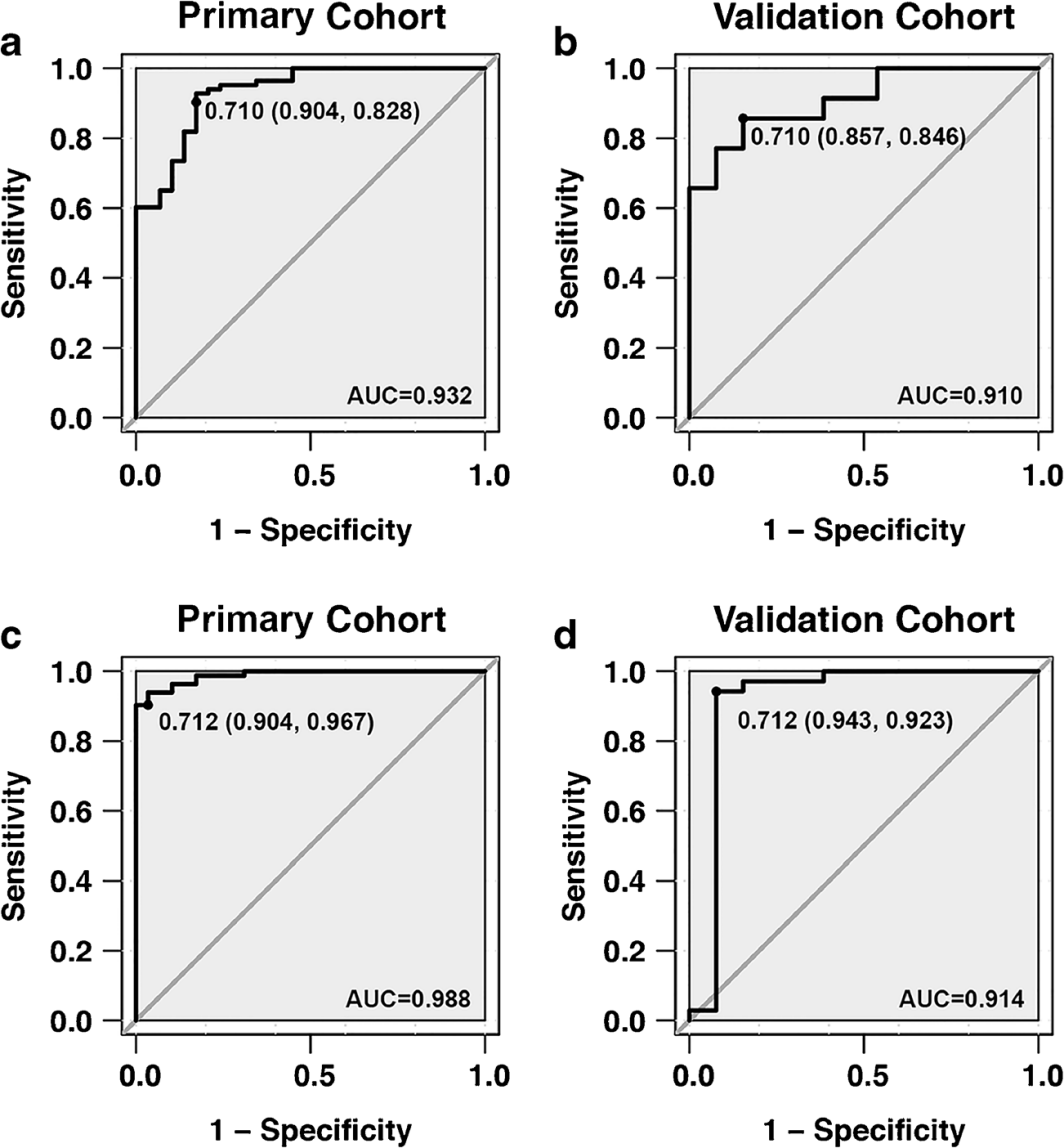
Sensitivity versus 1-specificity for the primary (**a**) and validation (**b**) cohorts using the diagnosis model based only on radiomics signatures and primary (**c**) and validation (**d**) cohorts for the integrated diagnosis model with both clinical features and radiomics signatures. The area under the curve (AUC) values are given, along with the threshold (sensitivity, specificity) for each case

**Fig. 5:**
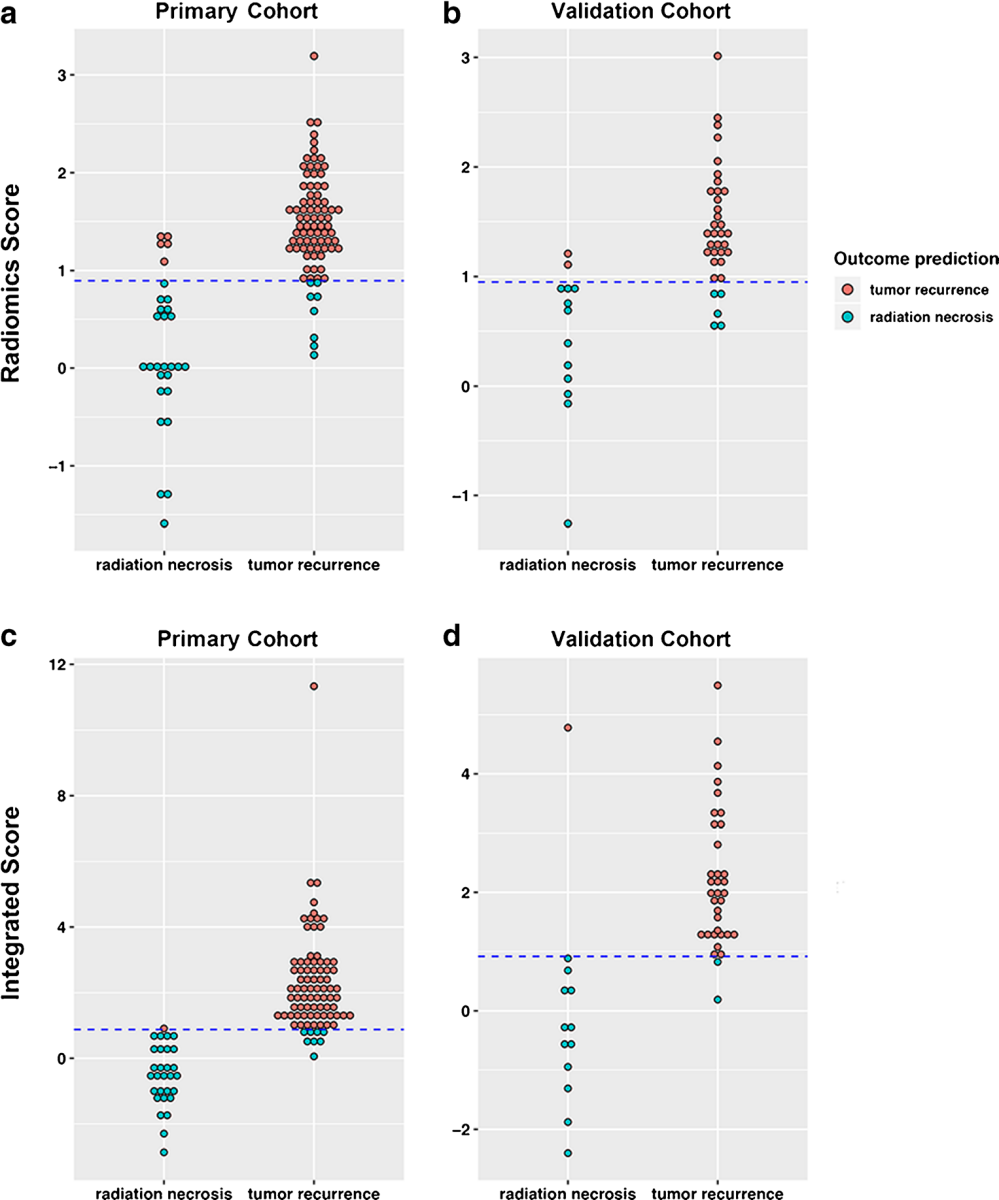
Diagnostic performance for the model based on only radiomics signature derived from ^18^F-FDG, ^11^C-MET, and MRI. Radiomics score for the primary (**a**) and validation (**b**) cohorts. Integrated score for the primary (**c**) and validation (**d**) cohorts. The diagrams show the differentiation ability of each model in terms of the agreement between the predicted risk and observed outcomes of tumor recurrence. The dotted line represents the threshold for tumor recurrence diagnosis: 0.895 and 0.905 for the radiomics score and integrated score, respectively

With a differential diagnosis threshold value of 0.710 for tumor recurrence and radiation necrosis, the model_[FDG+MET+MRI]_ yielded an AUC of 0.932 (95% CI, 0.887–0.986) in the primary cohort and 0.910 (95% CI, 0.855–0.973) in the validation cohort (Fig. 4a, b). In clinical diagnostic practice, this model demonstrated good diagnostic performance in distinguishing tumor recurrence in both primary and validation cohorts (Fig. 5a, b).

In addition, we further investigated and compared other three types of models using two of the three imaging modalities, i.e., model_[FDG+MET]_, model_[FDG+MRI]_, and model_[MET+MRI]_. The results of evaluation of the diagnostic performance by ROC analysis are presented in Table 2. The diagnostic accuracy of model_[FDG+MET+MRI]_ (AUC = 0.932; 95% CI = 0.887–0.986) was significantly higher than that of model[_MET+MRI]_ (AUC = 0.811; 95% CI = 0.711–0.912). However, although the AUC of model_[FDG+MET+MRI]_ was higher than those of the other two types of models (model_[FDG+MET]_: AUC = 0.898; 95% CI = 0.841–0.955 and model_[FDG+MRI]_: AUC = 0.891; 95% CI = 0.823–0.958), the differences were not statistically significant. In addition, the diagnostic performance of the models based on ^18^F-FDG, ^11^C-MET, and MRI, respectively, is provided in Table 3.

**Table 2:** Diagnostic performance of textural features in models with two imaging modalities

Modalities	^18^F-FDG + ^11^C-MET		^18^F-FDG + MRI		^11^C-MET + MRI	
Cohort	Primary	Validation	Primary	Validation	Primary	Validation
AUC	0.898	0.891	0.891	0.863	0.811	0.806
Accuracy	0.813	0.792	0.857	0.812	0.759	0.688
Sensitivity	0.781	0.750	0.854	0.833	0.780	0.722
Specificity	0.900	0.917	0.867	0.750	0.700	0.583
Threshold	0.749		0.711		0.740	
Feature number	13		15		17	

^*18*^*F-FDG*, ^18^F-fluorodeoxyglucose; ^*11*^*C-MET*, ^11^C-methionine; *MRI*, magnetic resonance imaging; *AUC*, area under the curve

**Table 3:** Diagnostic performance of textural features in single-modality model

Modalities	^18^F-FDG PET		^11^C-MET PET		MRI	
Cohort	Primary	Validation	Primary	Validation	Primary	Validation
AUC	0.868	0.810	0.767	0.750	0.699	0.622
Accuracy	0.784	0.714	0.721	0.735	0.694	0.691
Sensitivity	0.744	0.694	0.732	0.750	0.683	0.628
Specificity	0.897	0.769	0.690	0.692	0.744	0.651
Threshold	0.782		0.755		0.739	
Feature number	8		5		5	

^*18*^*F-FDG*, ^18^F-fluorodeoxyglucose; ^*11*^*C-MET*, ^11^C-methionine; *MRI*, magnetic resonance imaging; *AUC*, area under the curve

### Integrated diagnosis model

**Fig. 6:**
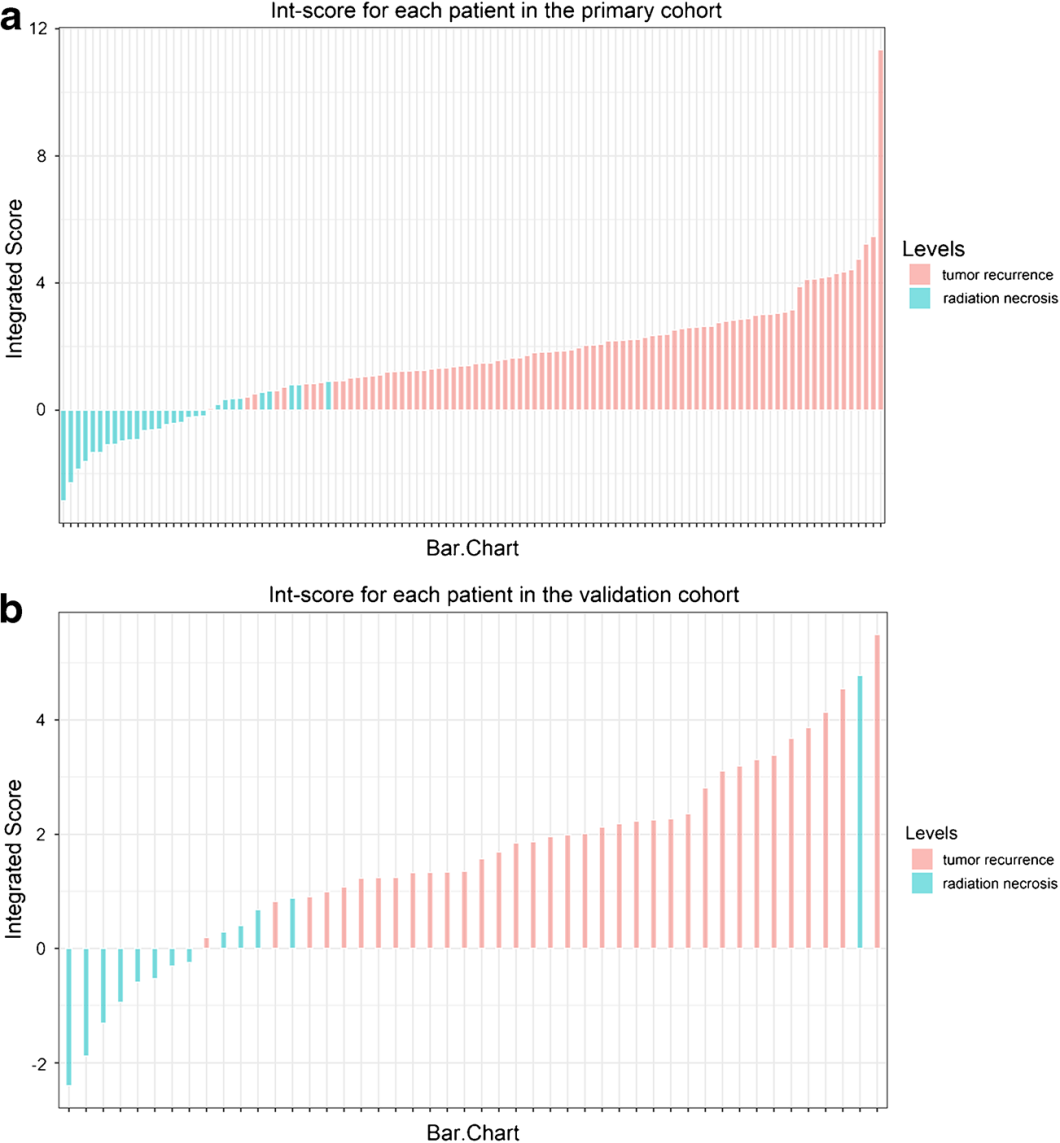
Integrated scores (int-score) distribution for all patients in the primary (**a**) and validation (**b**) cohorts, where the tumor recurrence (red bar) and radiation necrosis (green bar) confirmed by pathological results are indicated in different colors

Combined with clinical characteristics, we further developed an integrated diagnosis model by logistic regression. Finally, the age, TBR_mean_ of ^18^F-FDG PET, TBR_max_ of ^11^C-MET PET, and other 12 textual features were shown to be significant contributors for discriminating tumor recurrence from radiation necrosis (Supplemental Figure 1B). These features were included in the integrated score (int-score) calculation, along with the int-score distribution (Fig. 6).

Integrated score (int-score) calculation

$$ {\displaystyle \begin{array}{c}\mathrm{Int}-\mathrm{score}=1.55460\\ {}-0.06206\times \mathrm{age}\\ {}+0.11767\times {\mathrm{TBR}}_{\mathrm{mean}}\\ {}+1.17543\times {\mathrm{TBR}}_{\mathrm{max}}\\ {}+0.13864\times \mathrm{ClusterProminence}\_\mathrm{AllDirection}\_\mathrm{offset}4\_\mathrm{SD}\\ {}-0.24507\times \mathrm{ClusterShade}\_\mathrm{angle}135\_\mathrm{offfset}7\\ {}-0.19557\times \mathrm{InverseDifferenceMoment}\_\mathrm{AllDirection}\_\mathrm{offset}4\_\mathrm{SD}\\ {}-0.18425\times \mathrm{InverseDifferenceMoment}\_\mathrm{AllDirection}\_\mathrm{offset}7\_\mathrm{SD}\\ {}-0.11953\times \mathrm{InverseDifferenceMoment}\_\mathrm{angle}135\_\mathrm{offset}4\\ {}-0.16515\times \mathrm{ShortRunEmphasis}\_\mathrm{AllDirection}\_\mathrm{offset}4\_\mathrm{SD}\\ {}-0.01222\times \mathrm{ClusterProminence}\_\mathrm{angle}45\_\mathrm{offset}7\\ {}-0.29295\times \mathrm{HaralickCorrelation}\_\mathrm{AllDirection}\_\mathrm{offset}7\_\mathrm{SD}\\ {}+0.20089\times \mathrm{ShortRunHighGreyLevelEmphasis}\_\mathrm{AllDirection}\_\mathrm{offset}1\_\mathrm{SD}\\ {}+0.03032\times \mathrm{Quantile}0.025\\ {}+0.12080\times \mathrm{Correlation}\_\mathrm{angle}45\_\mathrm{offset}7\\ {}+0.02933\times \mathrm{ShortRunHighGreyLevelEmphasis}\_\mathrm{AllDirection}\_\mathrm{offset}4\_\mathrm{SD}\end{array}} $$

The difference in the int-score values between the tumor recurrence and radiation necrosis patients in the primary cohort was significant (*p* < 0.001), which was then confirmed in the validation cohort (*p* < 0.001). Patients with tumor recurrence generally had higher int-score values in both the primary and validation cohorts (Table 1).

Notably, the integrated model yielded the largest AUC of 0.988 (95% CI, 0.975–1.000) in the primary cohort and 0.914 (95% CI, 0.881–0.945) in the validation cohort (Fig. 4c, d). With a threshold of 0.712, the integrated model demonstrated better diagnostic performance between prediction and observation than that of the model_[FDG+MET+MRI]_ (Fig. 5c, d). Compared with the predictive models derived only from textural features, the integrated model was significantly better at distinguishing postoperative tumor recurrence from radiation necrosis in patients with gliomas.

**Fig. 7:**
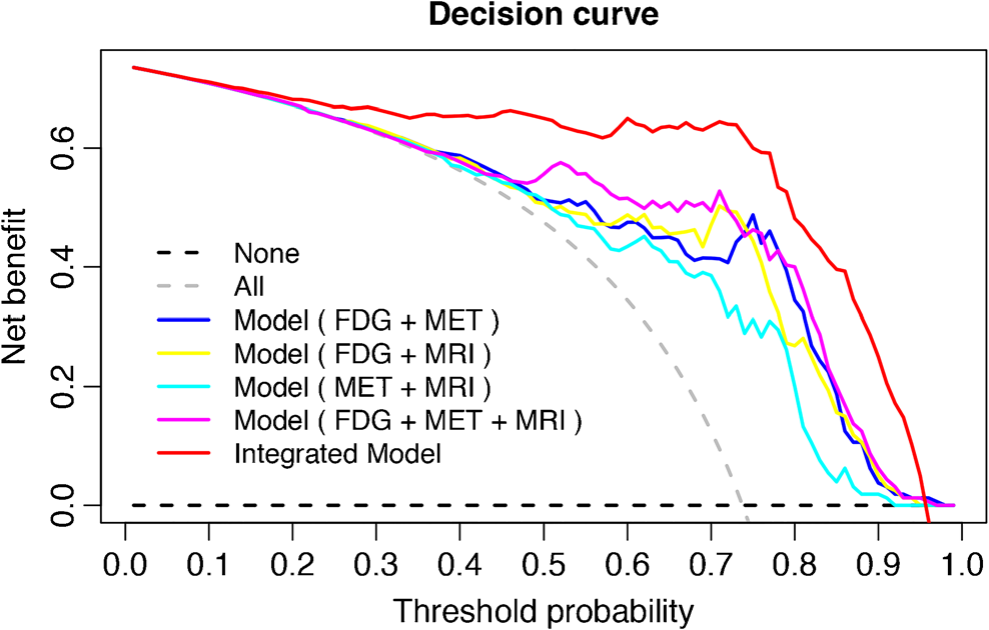
Decision curves (net benefit vs. threshold probability) for the integrated diagnosis model and the four models based on only radiomics signatures (^18^F-FDG, ^11^C-MET, and MRI). The gray dashed curve represents the assumption that all patients have tumor recurrence, while the black dashed curve line represents the assumption that no patients have tumor recurrence. The intersection of these curves at 0.741 indicates the point where the patients could receive the most net benefit from the integrated model

The decision curve for the integrated diagnosis model is compared with those of the other models (based only on radiomics signatures) in Fig. 7. The decision curve analysis showed that if the threshold probability of the patients was > 0.15, performing tumor recurrence diagnosis using the integrated diagnostic model added more benefit than either the treat-all-patients scheme or the treat-none scheme; with the optimal threshold of 0.741, the patients would receive the most benefit from clinical treatment.

## Discussion

In the present study, we developed and validated a radiomics signature–based diagnostic model for individualized discrimination of postoperative glioma recurrence from radiation necrosis. Incorporating the clinical factors and radiomics signatures into an integrated model could provide better assistance for the postoperative diagnosis of tumor recurrence.

The accurate differentiation between tumor recurrence and radiation necrosis in postoperative follow-up is crucial for decision-making regarding further clinical treatment, and has been investigated in many studies by comparing quantitative imaging parameters and advanced imaging processing methods [27–30]. To improve the diagnostic efficiency, the synergetic effect of multiparametric PET and MRI parameters was highlighted in previous studies. This indicates that the integrated ^18^F-FET or ^18^F-FDG PET/MRI analysis could assist in the management of glioma patients by timely and conclusive recognition of true tumor recurrence [9, 10, 31]. Being embedded in clinical practice, radiomics could provide a comprehensive quantification of imaging information. Papp et al. [32] proposed that survival prediction could be improved using computer-supported predictive models considering in vivo, ex vivo, and patient features.

Our integrated model demonstrated adequate discrimination between tumor recurrence and radiation necrosis in both primary and validation cohorts. As the difference between AUC values of the primary and validation cohorts was not statistically significant, we propose that the integrated model was robust for diagnosis and could be applied in the validation cohort. This suggests that multidimensional individual information might be a more promising approach for improving clinical management of glioma patients [33, 34]. Clinical physicians and radiologists could use our integrated diagnostic model (with radiomics signatures and clinical variables available postoperatively) to perform an individualized diagnosis of the risk of glioma recurrence, which follows the current trend of personalized medicine [16, 35].

The proposed use of the integrated diagnostic model is assisting clinical decision-making for postoperative glioma patients during the follow-up process. However, the recurrence diagnosis could not provide a specific level of discrimination, which is necessary for clinical practice [36, 37]. The decision curve analysis used to assess whether the radiomics-based integrated model could assist clinical treatment decisions provides further information about clinical consequences based on threshold probability, and quantifies the net benefit [35, 36].

Performance differences in between single modalities revealed that the diagnostic model based on only ^18^F-FDG PET image features had higher AUC that suggested a better differential diagnosis performance, followed by models based on ^11^C-MET and MRI in turn. Furthermore, when the combined differentiation power of two-modality models was considered, the model_[FDG+MET]_ still yielded a superior differential ability for tumor recurrence, compared with the model_[FDG+MRI]_ and model_[MET+MRI]_. As the most widely used radiotracer in clinical practice, ^18^F-FDG biological metabolism may incorporate more invisible image information of lesions compared with ^11^C-MET and MRI in the present study, which could potentially strengthen the crucial role of clinical utility of ^18^F-FDG PET. This information would be useful for clinicians to help optimize future diagnostic protocols for gliomas.

The repeatability radiomics model is of an important issue that could be affected by several factors, and image segmentation approaches are a common influencing factor. In our study, the ROIs were delineated manually that may not be favored in radiomics models. Although automated segmentation solutions may provide better support for the repeatability of radiomics results, accounting for clinical information not present in the images is beyond the capabilities of the automated method. In addition, the method to be chosen also depends on tumor type, involvement of neighboring structures, and image features [38]. Therefore, there is a need for active radiologist involvement in the segmentation process for both automated and semi-automated methods; moreover, automatically generated contours can be used only as a starting point for lesion delineation by the physician who may decide to modify them according to his/her knowledge [39].

The histological grade of the gliomas has been reported to be a predictor of patient prognosis [40–42]. Unexpectedly, the addition of the histologic grade to our integrated discrimination model did not improve the diagnostic performance, which may be attributed to the introduction of sampling bias due to the heterogenicity of glioma tissue, which may decrease the accuracy of the model. Therefore, the use of the radiomics signature, age, and uptake parameters of PET are recommended for tumor recurrence diagnosis with satisfactory discrimination.

Although *IDH1* mutation has remained an independent favorable prognostic molecular marker for gliomas, and is more objective and reliable than clinical criteria [43, 44], all malignant gliomas with various molecular characteristics have the possibility of recurrence after operation. In the present study, the integrated model could yield higher accuracy in tumor recurrence evaluation without the assistance of glioma-related molecular markers. Furthermore, it is speculated that the inclusion of molecular markers into the model may further enhance its diagnosis power.

There are some limitations in the present study. First, the sample size was relatively small for radiomics analysis, and further studies are required to verify the current findings. Second, the radiation necrosis group was relatively small for analysis, and the diagnostic thresholds of the integrated model may be cohort-specific; the results shall be carefully interpreted. Third, genetic characteristics, such as *IDH1* mutations, were not available for the whole cohort. In addition, the whole cohort was not divided by tumor histologic type for further stratification. However, our integrated diagnostic model is expected to assist and facilitate individualized postoperative discrimination of tumor recurrence from radiation necrosis in glioma patients.

## Conclusion

In conclusion, this paper presents an integrated model that incorporates both patient features and radiomics signature. The model presented can be conveniently used to facilitate postoperative individualized discrimination of tumor recurrence in glioma patients.

## Electronic supplementary material

ESM 1 (DOCX 276 kb)
