# Supplementary material for: Individualized discrimination of tumor recurrence from radiation necrosis in glioma patients using an integrated radiomics-based model
Source: Eur J Nucl Med Mol Imaging. 2019 Nov 26;47(6):1400–11. doi: 10.1007/s00259-019-04604-0 (PMC7188738; doi:10.1007/s00259-019-04604-0)
Supplement: Supplementary file 1 — (DOCX 276 kb) [file 259_2019_4604_MOESM1_ESM.docx]

**Supplementary Data**

**Appendix 1: The algorithms for radiomics feature extraction**

**Part I: Histogram parameters**

**1) Quantile 0.025**

Quantile normalization is a global adjustment method that assumes the same statistical distribution for each sample. The normalization is achieved by forcing the observed distributions to be the same, while the average distribution is obtained by taking averaging each quantile across the samples. The probability distributions of observations are divided into contiguous intervals with equal probabilities. For a finite population of *N* equally probable values indexed 1, ..., *N* from lowest to highest, the *k*-th *q*-quantile of the population is calculated using *Ip = Nk/q*. Here, we used five different quantiles: Quantile0.025, Quantile0.25, Quantile0.5, Quantile0.75, and Quantile0.975.

**2) Correlation_angle45_offset7**

Correlation measures the linear dependency of greyscale levels of neighboring pixels (i.e., similarity of the grey levels of neighboring pixels) over the entire selected image. A range of [-1,1] was used, where a correlation of 1 or -1 corresponded to a perfectly positively or negatively correlated image, respectively. The correlation angle was 45˚ and the offset was 7.

Formula:

$$\boldsymbol{-}\sum_{\boldsymbol{i,j}} \frac{\boldsymbol{(i-\mu)(j-\mu)g(i,j)}}{\sigma^{\boldsymbol{2}}}$$

$\boldsymbol{g}$ is the greyscale levels matrix；$\boldsymbol{\mu}\mathrm{is}$the mean gray level intensity of $\boldsymbol{g}\boldsymbol{；}\sigma\mathrm{is} the standard deviaon$of $\boldsymbol{g}\boldsymbol{；}\boldsymbol{i}$, $\boldsymbol{j}$ is the coordinate, $\boldsymbol{g(i,j)}$ representing the ($\boldsymbol{i}$, $\boldsymbol{j}$) elements of $\boldsymbol{g}$

**Part II: Texture Parameters**

**1) ClusterProminence_AllDirection_offset4_SD / ClusterProminence_angle45_**

**offset7**

Cluster prominence is a measure of asymmetry of a given distribution, where high values indicate that the symmetry of the image is low. In medical imaging, low values of cluster prominence represent a smaller peak for the image greyscale level, where the greyscale variance about the mean is lower.

Include:

(ClusterProminence_AllDirection_offset1,

ClusterProminence_AllDirection_offset1_SD,

ClusterProminence_angle0_offset1,

ClusterProminence_angle45_offset1,

ClusterProminence_angle90_offset1,

ClusterProminence_angle135_offset1,

ClusterProminence_AllDirection_offset4,

ClusterProminence_AllDirection_offset4_SD,

ClusterProminence_angle0_offset4,

ClusterProminence_angle45_offset4,

ClusterProminence_angle90_offset4,

ClusterProminence_angle135_offset4,

ClusterProminence_AllDirection_offset7, ClusterProminence_AllDirection_offset7_SD,

ClusterProminence_angle0_offset7,

ClusterProminence_angle45_offset7,

ClusterProminence_angle90_offset7,

ClusterProminence_angle135_offset7)

Formula:

$$\sum_{i,j} \left( \left( i-\mu\right)+\left( j-\mu\right) \right)^{4}g(i,j)$$

**2) ClusterShade_angle135_offset7**

Cluster analysis or clustering is a common technique for statistical data analysis, where a set of objects is grouped so that objects in the same cluster are more similar than those in other clusters. In the Cluster Shade algorithm, we grouped similar view samples according to their position and, optionally, normal into clusters. We used 36 parameters related to cluster analysis, where the following 18 are related to Cluster Shade.

(ClusterShade_AllDirection_offset1,

ClusterShade_AllDirection_offset1_SD,

ClusterShade_angle0_offset1,

ClusterShade_angle45_offset1,

ClusterShade_angle90_offset1,

ClusterShade_angle135_offset1,

ClusterShade_AllDirection_offset4,

ClusterShade_AllDirection_offset4_SD,

ClusterShade_angle0_offset4,

ClusterShade_angle45_offset4,

ClusterShade_angle90_offset4,

ClusterShade_angle135_offset4,

ClusterShade_AllDirection_offset7,

ClusterShade_AllDirection_offset7_SD,

ClusterShade_angle0_offset7,

ClusterShade_angle45_offset7,

ClusterShade_angle90_offset7,

ClusterShade_angle135_offset7).

Formula:

$$\sum_{i,j} \left( \left( i-\mu\right)+\left( j-\mu\right) \right)^{3}g(i,j)$$

**Part III:** Gray-level co-occurrence matrix **(GLCM) Parameters**

**1) InverseDifferenceMoment_AllDirection_offset4_SD/**

**InverseDifferenceMoment_AllDirection_offset7_SD/**

**InverseDifferenceMoment_angle135_offset4**

The inverse difference moment (IDM) describes the local homogeneity, where a high value indicates that the local grayscale level is uniform and the inverse GLCM is high. The IDM weight value is the inverse of the contrast weight.

Formula:

$$\sum\sum\frac{\boldsymbol{1}}{{\boldsymbol{1+}\left( \boldsymbol{i-j} \right)}^{\boldsymbol{2}}}\boldsymbol{g}\boldsymbol{(i,j)}$$

$\boldsymbol{g}$ is the normalized co-occurrence matrix; $\boldsymbol{i}$, $\boldsymbol{j}$ is the coordinate determined by offset and angle, $\boldsymbol{g(i,j)}$ representing the ($\boldsymbol{i}$, $\boldsymbol{j}$) elements of $\boldsymbol{g}$

**2) HaralickCorrelation_AllDirection_offset7_SD**

This algorithm measures the degree of similarity of the grayscale level of the image in the row or column direction. This value represents the correlation of the local greyscale level, where a higher value represents a better correlation.

(HaralickCorrelation_AllDirection_offset1,

HaralickCorrelation_AllDirection_offset1_SD,

HaralickCorrelation_angle0_offset1,

HaralickCorrelation_angle45_offset1,

HaralickCorrelation_angle90_offset1,

HaralickCorrelation_angle135_offset1,

HaralickCorrelation_AllDirection_offset4, HaralickCorrelation_AllDirection_offset4_SD,

HaralickCorrelation_angle0_offset4,

HaralickCorrelation_angle45_offset4,

HaralickCorrelation_angle90_offset4,

HaralickCorrelation_angle135_offset4,

HaralickCorrelation_AllDirection_offset7, HaralickCorrelation_AllDirection_offset7_SD,

HaralickCorrelation_angle0_offset7,

HaralickCorrelation_angle45_offset7,

HaralickCorrelation_angle90_offset7,

HaralickCorrelation_angle135_offset7)

Formula:

$$\boldsymbol{-}\sum_{\boldsymbol{i,j}} \frac{{\left( \boldsymbol{i,j} \right)\boldsymbol{g}\left( \boldsymbol{i,j} \right)\boldsymbol{-\mu}}_{\boldsymbol{t}}^{\boldsymbol{2}}}{\sigma_{\boldsymbol{t}}^{\boldsymbol{2}}}$$

**Part IV: Run-length matrix parameters**

The greyscale level run-length matrix (RLM) Pr(i,j|θ) is defined as the number of runs with pixels of grayscale level *i* and run length *j* for a given direction θ. RLM quantifies the grey level runs and were generated for each sample image segment with directions of 0°, 45°, 90°, and 135°. Then, the following ten statistical features were derived: short run emphasis, long run emphasis, grey level non-uniformity, run length non-uniformity, low grey level run emphasis, high grey level run emphasis, short run low grey level emphasis, short run high grey level emphasis, long run low grey level emphasis, and long run high grey level emphasis.

**1) ShortRunEmphasis_AllDirection_offset4_SD**

ShortRunEmphasis_AllDirection_offset1, ShortRunEmphasis_AllDirection_offset1_SD,

ShortRunEmphasis_angle0_offset1,

ShortRunEmphasis_angle45_offset1,

ShortRunEmphasis_angle90_offset1,

ShortRunEmphasis_angle135_offset1,

ShortRunEmphasis_AllDirection_offset4, ShortRunEmphasis_AllDirection_offset4_SD,

ShortRunEmphasis_angle0_offset4,

ShortRunEmphasis_angle45_offset4,

ShortRunEmphasis_angle90_offset4,

ShortRunEmphasis_angle135_offset4,

ShortRunEmphasis_AllDirection_offset7, ShortRunEmphasis_AllDirection_offset7_SD,

ShortRunEmphasis_angle0_offset7,

ShortRunEmphasis_angle45_offset7,

ShortRunEmphasis_angle90_offset7,

ShortRunEmphasis_angle135_offset7

Formula:

$$\frac{\boldsymbol{1}}{\boldsymbol{n}_{\boldsymbol{r}}}\sum_{\boldsymbol{i=1}}^{\boldsymbol{M}} \sum_{\boldsymbol{j=1}}^{\boldsymbol{N}} \frac{\boldsymbol{p(i,j,}\boldsymbol{\theta}\boldsymbol{)}}{\boldsymbol{j}^{\boldsymbol{2}}}$$

**2) ShortRunHighGreyLevelEmphasis_AllDirection_offset1_SD / ShortRunHighGreyLevelEmphasis_AllDirection_offset4_SD**

Formula:

$$\boldsymbol{SRHGE}\left( \boldsymbol{\theta} \right)\boldsymbol{=}\frac{\boldsymbol{1}}{\boldsymbol{n}_{\boldsymbol{r}}}\sum_{\boldsymbol{j=1}}^{\boldsymbol{N}} \sum_{\boldsymbol{i=1}}^{\boldsymbol{M}} \frac{\boldsymbol{p(i,j,\theta)i}^{\boldsymbol{2}}}{\boldsymbol{j}^{\boldsymbol{2}}}$$

**
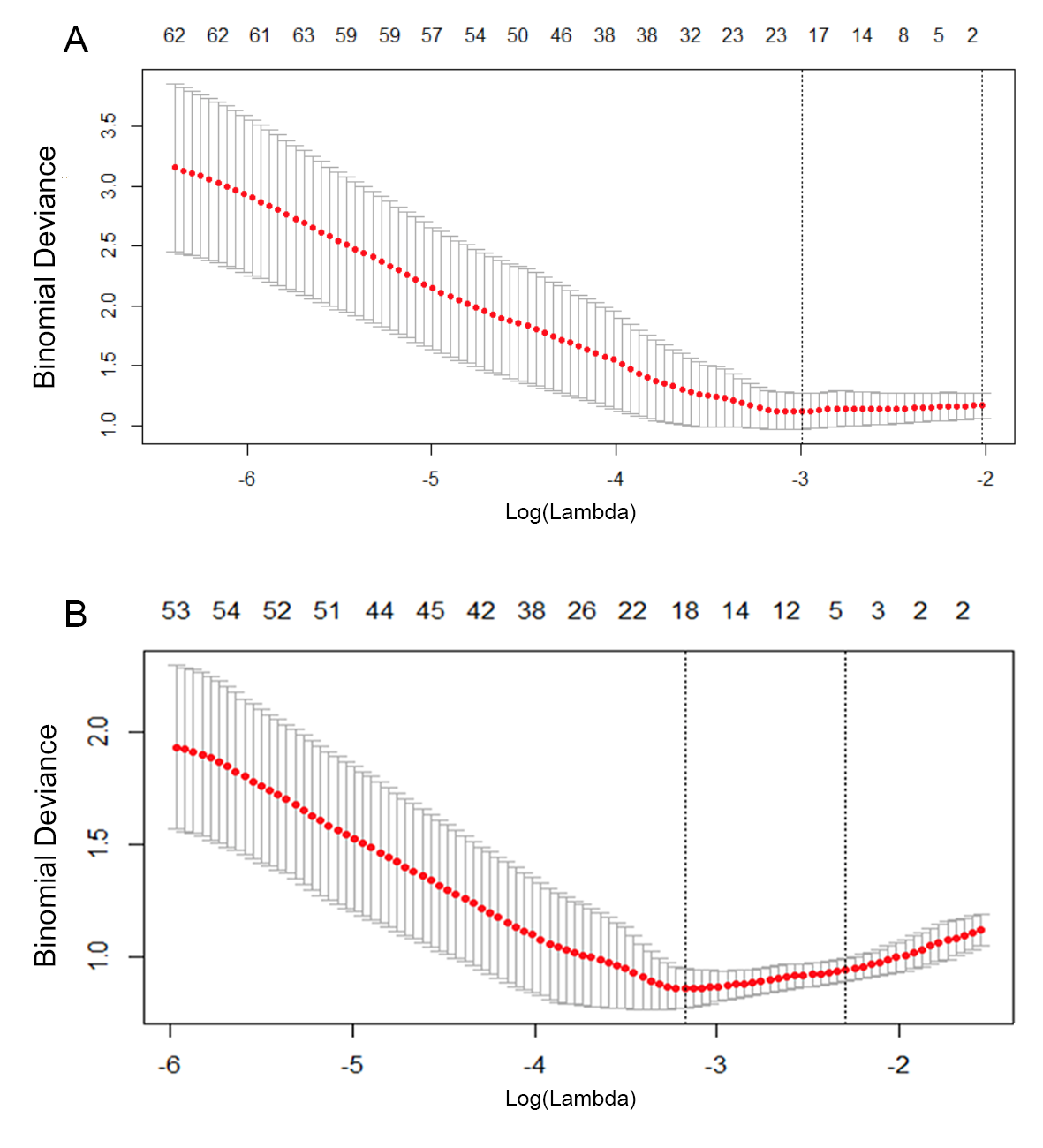
**

**Supplemental Figure 1.** (A) Texture feature selection and (B) clinical and texture feature selection using the LASSO binary logistic regression model. We used cross-validation via minimum criteria to select the tuning parameter (λ). The binomial deviance is plotted versus log (λ). The dotted vertical lines indicate the optimal values using the minimum criteria and the one-standard-error of the minimum criteria. The cross-validation method gave λ values of 0.050 and 0.042 for (A) and (B), respectively.

**Supplemental Table 1.** Comparison of patient features of the primary and validation cohorts (n = 160).

| Characteristics | Primary cohort (n = 112) | Validation cohort (n = 48) | | *p** |
| --- | --- | --- | --- | --- |
| Age, mean ± SD (y) | 44.54 ± 10.38 | 44.58 ± 11.79 | 0.980 | |
| Gender, No. (%) |  |  | 0.944 | |
| Male | 67 (59.8) | 29 (60.4) |  | |
| Female | 45 (40.2) | 19 (39.6) |  | |
| MRI contrast enhancement, No. (%) |  |  | 0.873 | |
| Yes | 99 (88.4) | 42 (87.5) |  | |
| No | 13 (11.6) | 6 (12.5) |  | |
| ^18^F-FDG uptake |  |  |  | |
| TBR_max_ | 3.67 ± 2.51 | 3.93 ± 2.77 | 0.550 | |
| TBR_mean_ | 2.50 ± 1.45 | 2.66 ± 1.66 | 0.541 | |
| ^11^C-methionine uptake |  |  |  | |
| TBR_max_ | 3.54 ± 2.55 | 3.58 ± 1.94 | 0.927 | |
| TBR_mean_ | 2.40 ± 1.97 | 2.30 ± 1.21 | 0.731 | |
| WHO Grade, No. (%) |  |  | 0.114 | |
| II | 56 (50.0) | 16 (33.3) |  | |
| III | 27 (24.1) | 18 (37.5) |  | |
| IV | 29 (25.9) | 14 (29.2) |  | |
| Radiomics score, mean ± SD | 1.15 ± 0.82 | 1.18 ± 0.74 | 0.840 | |
| Integrated score, mean ± SD | 1.54 ± 1.86 | 1.58 ± 1.69 | 0.914 | |

* *p* values derived from univariable association analysis between clinical primary and validation cohorts. SD: standard deviation; MRI: magnetic resonance imaging; FDG: fluorodeoxyglucose; TBR: tumor-to-background ratio

**Supplemental Table 2.** Comparison of patient features between the primary and validation cohorts for the tumor recurrence cohort (n = 118).

| Characteristics | Primary cohort (n = 83) | Validation cohort (n = 35) | | *p** |
| --- | --- | --- | --- | --- |
| Age, mean ± SD (years) | 43.87 ± 9.90 | 45.94 ± 11.26 | 0.320 | |
| Gender, No. (%) |  |  | 0.790 | |
| Male | 50 (60.2) | 22 (62.9) |  | |
| Female | 33 (39.8) | 13 (37.1) |  | |
| MRI contrast enhancement, No. (%) |  |  | >0.999 | |
| Yes | 76 (91.6) | 32 (91.4) |  | |
| No | 7 (8.4) | 3 (8.6) |  | |
| ^18^F-FDG uptake |  |  |  | |
| TBR_max_ | 4.15 ± 2.41 | 4.53 ± 2.96 | 0.465 | |
| TBR_mean_ | 2.83 ± 1.38 | 3.04 ± 1.75 | 0.498 | |
| ^11^C-methionine uptake |  |  |  | |
| TBR_max_ | 4.17 ± 2.62 | 4.15 ± 1.53 | 0.965 | |
| TBR_mean_ | 2.81 ± 2.12 | 2.65 ± 1.07 | 0.680 | |
| WHO Grade, No. (%) |  |  | 0.221 | |
| II | 38 (45.8) | 11 (31.4) |  | |
| III | 21 (25.3) | 14 (40.0) |  | |
| IV | 24 (28.9) | 10 (28.6) |  | |
| Radiomics score, mean ± SD | 1.49 ± 0.52 | 1.46 ± 0.55 | 0.790 | |
| Integrated score, mean ± SD | 2.27 ± 1.53 | 2.20 ± 1.18 | 0.816 | |

* *p* values derived from univariable association analysis between clinical primary and validation cohorts. SD: standard deviation; MRI: magnetic resonance imaging; FDG: fluorodeoxyglucose; TBR: tumor-to-background ratio

**Supplemental Table 3.** Comparison of patient features between the primary and validation cohorts for the radiation necrosis cohort (n = 42).

| Characteristics | Primary cohort (n = 29) | Validation cohort (n = 13) | | *p** |
| --- | --- | --- | --- | --- |
| Age, mean ± SD (years) | 46.45 ± 11.61 | 40.92 ± 12.84 | 0.175 | |
| Gender, No. (%) |  |  | 0.773 | |
| Male | 17 (58.6) | 7 (53.8) |  | |
| Female | 12 (41.4) | 6 (46.2) |  | |
| MRI contrast enhancement, No. (%) |  |  | >0.999 | |
| Yes | 23 (79.3) | 10 (76.9) |  | |
| No | 6 (20.7) | 3 (23.1) |  | |
| ^18^F-FDG uptake |  |  |  | |
| TBR_max_ | 2.28 ± 2.29 | 2.32 ± 1.16 | 0.952 | |
| TBR_mean_ | 1.54 ± 1.21 | 1.63 ± 0.73 | 0.794 | |
| ^11^C-MET uptake |  |  |  | |
| TBR_max_ | 1.74 ± 1.05 | 2.05 ± 2.14 | 0.540 | |
| TBR_mean_ | 1.23 ± 0.62 | 1.33 ± 1.06 | 0.703 | |
| WHO Grade, No. (%) |  |  | 0.357 | |
| II | 18 (62.1) | 5 (38.4) |  | |
| III | 6 (20.7) | 4 (30.8) |  | |
| IV | 5 (17.2) | 4(30.8) |  | |
| Radiomics score, mean ± SD | 0.19 ± 0.78 | 0.43 ± 0.68 | 0.350 | |
| Integrated score, mean ± SD | -0.52 ± 0.95 | -0.09 ± 1.76 | 0.308 | |

* *p* values derived from univariable association analysis between clinical primary and validation cohorts. SD: standard deviation; MRI: magnetic resonance imaging; ^18^F-FDG: ^18^F-fluorodeoxyglucose; ^11^C-MET: ^11^C-methionine; TBR: tumor-to-background ratio
